# Medical Botany

**Published:** 1834-07-01

**Authors:** 


I. HORTUS MEDICUS; OR FIGURES AND DESCRIPTIONS OF THE MORE IMPORTANT PLANTS USED IN MEDICINE, &c. By *George Graves*. Quarto, pp. 264, 44 Plates. Edinburgh, 1834.

II. MEDICAL BOTANY. By *John Stephenson*, M.D. and *J. M. Churchill*, F.L.S. New Edition, by *Gilbert Burnett*, Professor of Botany in King's College, London. Octavo, London.

THE former of these publications is a very splendid and admirable work. The plates are excellent—some of surpassing beauty, and all most faithfully correct. The figures of the "arum maculatum," "menyanthes trifoliata," "secale cornutum," "amanita muscaria," &c. are quite perfect. To all who are engaged in the delightful pursuits of botany, and who feel an interest in the promotion of the pictorial art, we recommend this volume with confidence and pleasure; and we are the more anxious to do so, as the nature of its contents prevents us from making such extracts as might enable our readers thereby to judge for themselves. The botanical description of each plant is given with much accuracy, and the remarks on the medical properties and uses, drawn up by Dr. Davie Morries, are sufficiently explicit and instructive. Take, for example, his account of the secale cornutum.

"In many parts of continental Europe, the rye forms a principal part of the food of the inhabitants; in some seasons and in particular districts the grain is subject to a disease which renders it highly poisonous to men and animals. Some animals do not seem easily affected by it; others, among which are swine, geese, fowls, &c. are affected with diarrhœa, vertigo, and latterly with suppurating tumours and gangrene. Two distinct diseases are caused by the habitual use of the ergotized grain. One, the *Ergotisme convulsive* of the French; the other, the *Gangrene sèche* of the same authors. The first form of the disease commences with vertigo, dimness of sight, and loss of feeling, followed by cramps and convulsions of the whole body, *risus sardonicus*, yellowness of the countenance, excessive thirst, excruciating pains in the limbs, and dull, small, and imperceptible pulse. When the symptoms are of this aggravated nature, the disease generally proves fatal in from twenty-four to forty-eight hours. In milder forms, the convulsions come on in paroxysms, and are preceded for some time by lassitude and the feeling of insects crawling over the surface of the body; in the intervals the appetite is voracious. The pulse and excretions are natural; the disease either terminates in recovery, preceded by scattered suppurations, cutaneous eruptions, anasarca, and diarrhœa, or it ends fatally amidst prolonged sopor and convulsions. The next form of the disease,—the dry gangrene, commences with general uneasiness, weakness, and a feeling of insects crawling over the skin. When these symptoms have continued for some days or weeks, the extremities become cold, stiff, white, and benumbed, and so insensible that deep incisions are not felt; excruciating pains supervene, with fever, headach, and bleeding from the nose; finally, the affected parts gradually shrivel and drop off by the joints; healthy granulations succeed, but the system is frequently so worn out that the person dies before this favourable change occurs. The appetite continues voracious throughout. Various other modifications of this formidable epidemic have been observed in Germany and Switzerland. From the improved state of agriculture, the disease is becoming more rare, though some cases have occurred in Germany since the commencement of the present century. Another very peculiar property has been attributed to the ergot of rye; it is that of exciting the action of the uterus when dormant from protracted unsuccessful efforts to expel the child. This property is supported by the testimony of many accoucheurs; but it is a remedy which ought to be given only in urgent cases, and in the most careful and guarded manner. It has been said to have the power of causing abortion; but it is the opinion of the best authorities, that it only possesses the property of increasing the action of the uterus when it has already commenced, and that it has no power of inducing uterine action in the early months of pregnancy, at least not without causing constitutional disturbance of a very dangerous nature.* The cause of the ergot is not ascertained; it is by some supposed to arise from the puncture of an insect, by others it is said to arise from the presence of parasitical fungi." 128.

Describing the colchicum autumnale, he observes—

"Colchicum possesses the remarkable property of increasing the quantity of uric acid in the urine. This was first observed by Professor Chelius, who states that the quantity of uric acid is nearly doubled after the Colchicum has been taken for twelve days.† As an increased discharge of uric acid immediately precedes or accompanies the favourable termination of a gouty paroxysm, and as the concretions found in the joints of gouty persons are composed of uric acid in union with ammonia, it seems to me not an unnatural conclusion, that the property of increasing the discharge of the acid is as likely to be the cause of the good effects of the Colchicum, as either its sedative or cathartic qualities." 130.

To the lover of botany, the following observations of Mr. Graves, on the presence of sugar in many of our indigenous and most common grasses, will be interesting.

"It is sometimes to be found in a crystalline form, and nearly pure, in the stems and roots of some of our native grasses; as the *Poa aquatica, Poa fluitans*, and *Catabrosa aquatica*. It is not a little remarkable that the British species of grass in which sugar is found in a crystalline form, are entirely aquatic; I first met with it in this state in *Poa aquatica*. The circumstance which led me to discover it, was, observing some swine eagerly rooting among mud and weeds that were thrown out of a water-course. I remarked they greedily devoured the roots and stems of some species of grass, which induced me to examine the kind, which proved to be those of the *Poa aquatica*. The roots were large and succulent, but having been exposed to the action of the sun and air for some days, the outer parts of the stems and roots had become dry. I peeled one or two of these, and at the base of each leaf, and of the sheaths covering the root, I found some small transparent crystals, which were pure sugar. I obtained nearly half an ounce of these, and have since found that this plant, as well as the other two above named, which are the sweetest of our native grasses, if removed from the watery spots in which they constantly grow, and exposed to the action of the sun and air, produce small grains of sugar as before-mentioned. Several pieces of Fucus or sea-weed, also spontaneously exude sugar." 69.

With one more extract we shall conclude.

"Some of the species in this and other genera of the natural order "aroideæ," possess, even in a more remarkable degree than the *Arum maculatum*, the property of causing painful swelling of the tongue. Dr. Hooker mentions the case of a gardener, who, from merely tasting a bit of the *Caladium Seguinum* (Dumb-Cane), was confined to the house for several days by swelling of the tongue, accompanied with excruciating pain. The juice of this last mentioned

* "The above account is taken from the article Ergot in Dr. Christison's work on Poisons."

† Richard, Histoire Naturelle Medicale, I. 357.

plant, as well as that of the *Arum ovatum*, is sometimes used by sugar manufacturers to assist the granulation when the juice is too viscid. The acrimony of the Arum is entirely destroyed by heat, and though, as has been already observed, a virulent poison when fresh, it becomes not only harmless, but even nutritious when properly prepared. The inhabitants of the Isle of Portland manufacture the fecula and send it to London, where it is sold under the name of Portland Sago. Culture seems to have the power of destroying the acrimony of this genus, and one of the species, the *Arum Colocosia* is grown as a pot-herb."* 8.

In taking leave of this work, we consider it our duty to point out to the authors what seem to us the chief errors they have fallen into; they are errors partly of commission and partly of omission. On comparing the botanical and pharmaceutical descriptions with those given by Drs. Duncan and A. T. Thomson, in their respective Dispensatories (books which are in every professional man's library), we find few or no new facts, and certainly no additional information as to the medical uses and properties of the plants. It may be said that the latter are so good as to leave no room for amplification. True; and what then? Instead of repeating the statements of others, we should have recommended the publication of the present very admirable engravings alone, as illustrative of those works on Materia Medica which have, for so many years, been established as leading authorities. Had Mr. Graves followed this plan, the Hortus Medicus might have been published at a cheaper rate, and very probably have obtained, in consequence, a wider circulation.

The omission of figures of many of the most important exotic plants is much to be regretted. In the place of the "anagallis arvensis," "arctium lappa," "helleborus fœtidus," &c. which are scarcely admitted into the catalogue of medical botany, it would have been wise to have substituted drawings of the plants which yield the croton oil, strychnine, jalap, ipecacuan, and cubebs; and assuredly the papaver somniferum ought to have been selected in preference to the p. rhœas, and the helleborus niger to the h. fœtidus. This defect we hope to see remedied in a subsequent volume; and the value of it, as well as of the present one, would be enhanced, if some method was followed in the arrangement of the plates. Let it be either alphabetical, botanical (this would be the best), or pharmaceutical.

The other work on Medical Botany, mentioned at the head of this article, has already obtained the stamp of a widely-extended approbation—and very justly so. It is published in monthly numbers, each containing four engravings, with descriptive letter-press; and the present edition has been much improved under the able superintendence of Mr. Burnett, Professor of Botany in King's College. Eighteen numbers have already (June) appeared, and the later ones fully merit the praise which we awarded to their earlier brethren. When completed, the work will possess this great advantage over the Hortus Medicus—that it will present an entire catalogue of figures of every vegetable admitted into the materia medica; and will thus afford to medical men a valuable book of reference, not only for the indigenous, but also for the exotic medicinal plants, in the event of their visiting foreign

* Rich. Hist. Nat. Med.

countries. So high is our opinion of its merits, that we recommend every student at college, and every surgeon who goes abroad, to have a copy, as one of the essential constituents of his library.

